# Emergency department revisits and hospital readmissions following a fall-related emergency department presentation in older adults: a systematic review and meta-analysis

**DOI:** 10.1093/ageing/afag271

**Published:** 2026-09-14

**Authors:** Chris Larue, Lotte Jonkman, Bjorn Winkens, Sofie Jansen, Nathalie van der Velde, Gideon H P Latten, Bart Spaetgens

**Affiliations:** Department of Internal Medicine, Section of Geriatric Medicine, Maastricht University Medical Centre+, Maastricht, Netherlands; Department of Emergency Medicine, Maastricht University Medical Centre+, Maastricht, Netherlands; Department of Internal Medicine, Section of Geriatric Medicine, Maastricht University Medical Centre+, Maastricht, Netherlands; Department of Emergency Medicine, Maastricht University Medical Centre+, Maastricht, Netherlands; Department of Methodology and Statistics, Maastricht University, Maastricht, Netherlands; Care and Public Health Research Institute (CAPHRI), Maastricht University, Maastricht, Netherlands; Department of Internal Medicine, Section of Geriatric Medicine, Amsterdam University Medical Centre, University of Amsterdam, Amsterdam, Netherlands; Aging & Later Life, Amsterdam Public Health research institute, Amsterdam, Netherlands; Department of Geriatric Medicine, Dijklander Hospital, Hoorn, the Netherlands; Department of Internal Medicine, Section of Geriatric Medicine, Amsterdam University Medical Centre, University of Amsterdam, Amsterdam, Netherlands; Aging & Later Life, Amsterdam Public Health research institute, Amsterdam, Netherlands; Department of Emergency Medicine, Maastricht University Medical Centre+, Maastricht, Netherlands; Department of Internal Medicine, Section of Geriatric Medicine, Maastricht University Medical Centre+, Maastricht, Netherlands; Care and Public Health Research Institute (CAPHRI), Maastricht University, Maastricht, Netherlands

**Keywords:** falls, older adults, emergency department revisits, hospital readmissions, acute care utilisation, frail older people, systematic review

## Abstract

**Background:**

Falls are a leading cause of emergency department (ED) visits in older adults, yet subsequent trajectories of acute care use remain poorly characterised. We aimed to estimate pooled proportions of ED revisits and hospital readmissions at multiple follow-up intervals within 1 year.

**Methods:**

In this systematic review and meta-analysis (PROSPERO: CRD420251167449), we searched PubMed, Embase and Scopus from inception to 30 November 2025 for observational studies including adults aged ≥65 years presenting to the ED after an index fall. Eligible studies reported ED revisits and/or hospital readmissions within 1 year. Two reviewers independently screened studies, extracted data and assessed risk of bias using the Newcastle–Ottawa Scale. Random-effects meta-analyses of proportions were conducted for each follow-up interval.

**Results:**

Nineteen studies (20 publications) were included, with overall low to moderate risk of bias. The cumulative proportions of patients experiencing an ED revisit increased with longer follow-up, from 3.9% within 3 days to 8.8% within 7 days, 15.0% within 30 days and 21.7% within 90 days (single study), reaching 37.5% at 6 months and 41.3% at 12 months. Hospital readmissions similarly increased from 10.4% at 30 days to 30.4% at 6 months and 31.9% at 12 months (single study). Substantial heterogeneity was observed across several pooled analyses.

**Conclusion:**

A fall-related ED presentation is associated with substantial cumulative acute care use over the following year. Falls may represent an important marker of broader vulnerability and ongoing healthcare needs. Future research should identify patients at high risk of recurrent acute care use and determine whether targeted follow-up reduces recurrent ED visits and hospital readmissions.

## Graphical Abstract

**Graphical Abstract:**
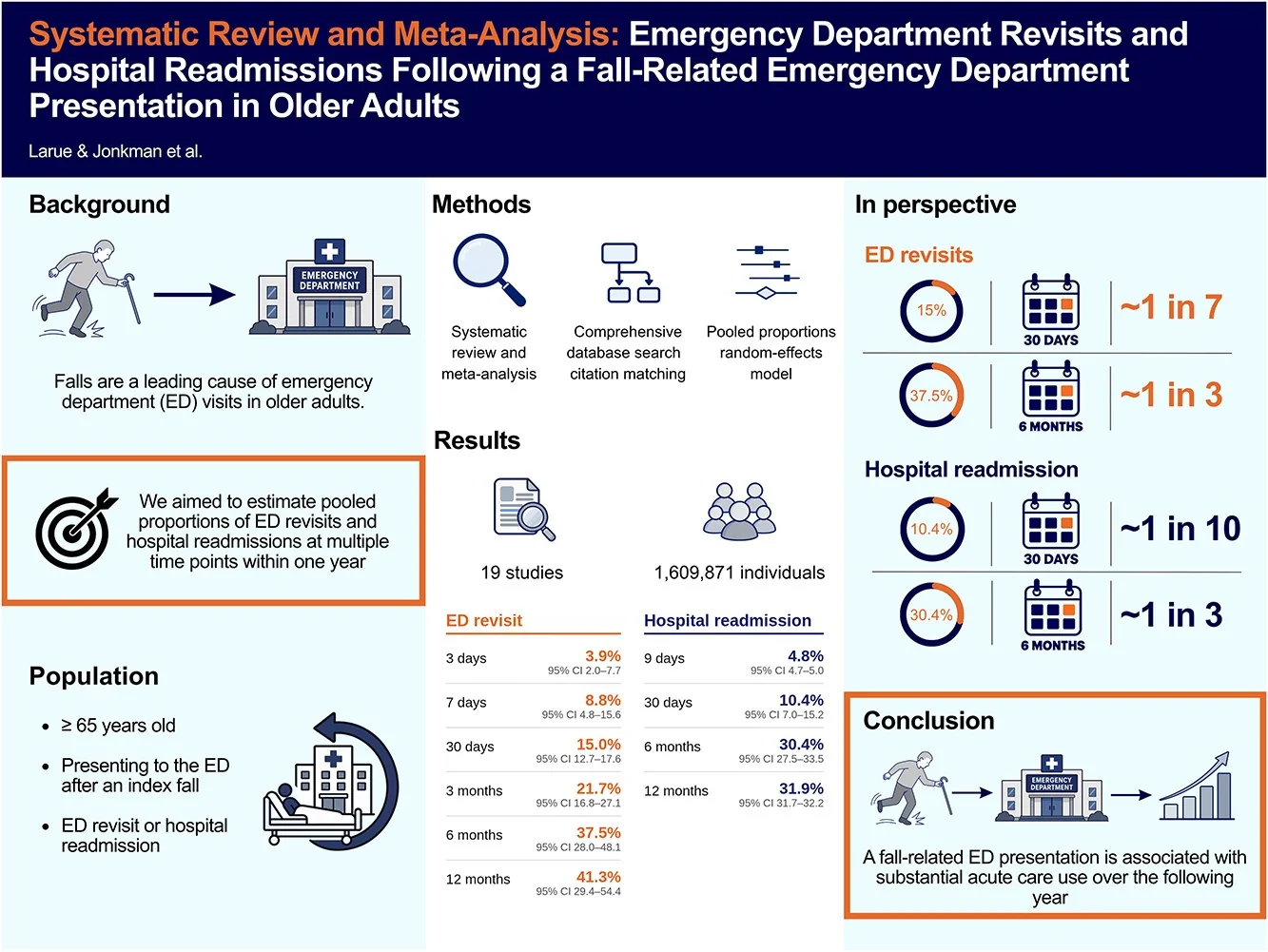

## Key points

- This systematic review quantifies recurrent acute care use following fall-related ED presentations in older adults.
- The cumulative proportion of recurrent acute care use exceeds 40% within 1 year.
- Falls may represent an important clinical marker of broader vulnerability and ongoing healthcare needs.
- Future research should evaluate targeted follow-up strategies and integrated models of care.

## Introduction

Falls are a leading cause of emergency department (ED) visits and unplanned hospital admissions in older adults and are challenging to manage appropriately [1]. Beyond causing injury, a fall can represent the first visible manifestation of impaired physiological resilience, frailty, multimorbidity and underlying (unidentified) diseases and disorders [2, 3]. Despite increasing attention to this issue, a substantial proportion of older adults revisit the ED or require hospital admission shortly after an index fall. As populations age, fall-related ED presentations are increasingly shaping both acute care demand and longer-term health trajectories [4].

Up to one in five older adults revisits the ED within 30 days after an index fall-related presentation, highlighting that recurrent healthcare use is common following a fall-related ED presentation and that such events are often not self-limiting [5]. Subsequent trajectories frequently extend beyond the ED, involving multiple specialties and care settings, and are associated with increasing care dependency, recurrent hospital admissions, progressive loss of intrinsic capacity and declining quality of life [6]. Accordingly, a fall-related ED presentation may represent an early marker of future adverse outcomes and a manifestation of potentially underlying (urgent) diseases and disorders rather than an isolated event. More broadly, falls may be viewed as clinical manifestations of frailty, multimorbidity, reduced intrinsic capacity and declining physiological resilience in older adults. As such, recurrent healthcare utilisation following a fall may reflect not only the consequences of the fall itself, but also the broader vulnerability of the individual.

An index fall-related ED presentation also represents an opportunity to identify older adults at high risk of recurrent falls and health-care use [7, 8]. However, most patients discharged after a fall do not receive guideline-recommended fall prevention care [9], and existing guidelines are often poorly aligned with the time constraints of the ED [8, 10]. Consequently, this window of opportunity is frequently underused, while evidence suggests that uptake of fall prevention is higher when patients are approached directly at the ED rather than later by telephone [11]. Robust quantification of recurrent health-care use is therefore needed to characterise post-fall trajectories and to inform early risk assessment and treatment after falls [12].

Data on ED revisits and hospital readmissions after a fall-related ED presentation remain fragmented and heterogeneous across studies and follow-up intervals. To our knowledge, no comprehensive synthesis has quantified these outcomes across both short-term and longer-term follow-up within the first year after a fall. We therefore aimed to estimate pooled proportions of ED revisits and hospital readmissions at multiple timepoints following a fall-related ED presentation in older adults.

## Methods

A systematic review and meta-analysis of observational studies including adults aged 65 years or older presenting to the ED after an index fall was conducted, with the aim of estimating ED revisit and hospital readmission rates. In addition, we descriptively summarised factors reported to be associated with these outcomes. The review was conducted and reported in accordance with Preferred Reporting Items for Systematic reviews and Meta-Analyses (PRISMA) 2020 guidelines [13] and registered in PROSPERO (CRD420251167449). Ethical approval was not required because only published data were used.

We searched PubMed (National Center for Biotechnology Information (NCBI) interface), Embase (Ovid platform) and Scopus (Elsevier platform) from database inception to 30 November 2025, without language restrictions. Search strategies combined controlled vocabulary (MeSH and Emtree) and free-text terms related to falls, older adults, ED care and revisit or readmission outcomes. Citation tracking and manual screening were used during search development. Full search strategies are provided in Supplementary Table S1.

**Figure 1:**
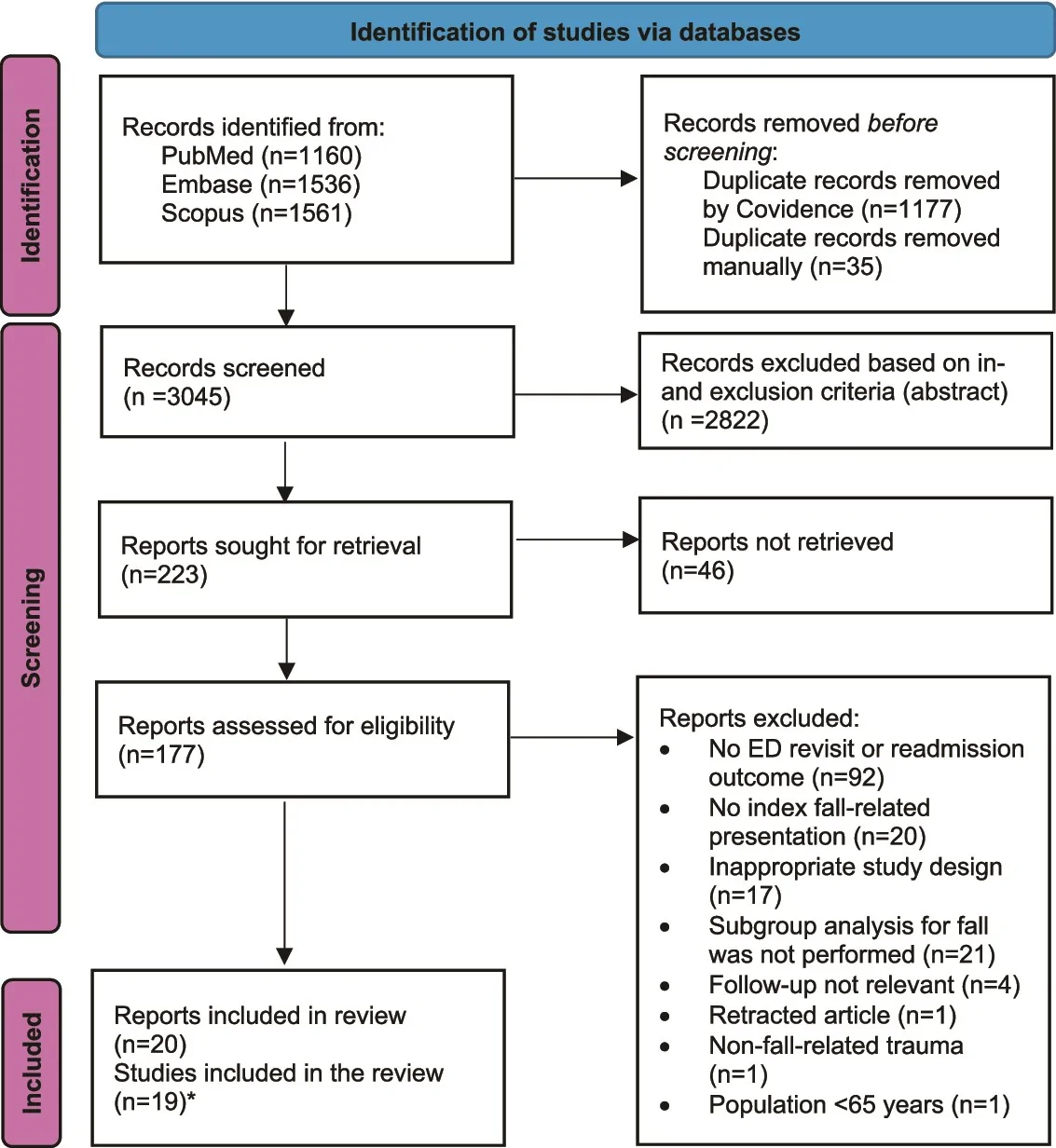
PRISMA 2020 flow diagram of study selection. ^*^Twenty reports representing 19 unique studies were included. Two reports described the same study population and were treated as a single study during data extraction and synthesis.

Records were imported into Covidence for deduplication and screening. Two reviewers independently screened titles and abstracts and assessed full texts for eligibility. One non-English full-text article required translation for eligibility assessment and data extraction. Translation was performed using ChatGPT (OpenAI) and subsequently verified against the original publication, including verification of all relevant text, tables and numerical data. Disagreements were resolved by discussion with two clinical supervisors and senior authors. The selection process is shown in a PRISMA flow diagram (Figure 1).

Prospective and retrospective observational studies were eligible, including cohort, registry-based, case–control and retrospective chart review studies. Case–control studies were considered eligible when cases and controls originated from a defined cohort of older adults presenting to the ED after a fall-related ED presentation and provided data on subsequent ED revisits, hospital readmissions and/or associated factors. Studies with mixed-age populations were included if data for adults aged 65 years or older were reported separately. We excluded studies of non-fall-related trauma, studies without post-ED follow-up and case reports, case series or cross-sectional studies. Studies were grouped by outcome (ED revisit or hospital readmission) and follow-up duration, up to 1 year after the index fall-related ED presentation.

### Data extraction and risk of bias

Two reviewers independently extracted data using a standardised, piloted form. Extracted variables included author, year, country, study design, setting, sample size, age, outcome measures and key results. Disagreements were resolved by consensus. When event counts were not reported, they were derived from proportions and denominators where possible and rounded to the nearest whole number. Missing or unclear data were requested from corresponding authors; studies were excluded if data remained unavailable.

Risk of bias was assessed independently using the Newcastle-Ottawa Scale (NOS) [14]. Consistent with previous systematic reviews, NOS scores were interpreted as high risk of bias (0–3 stars), moderate risk of bias (4–6 stars) and low risk of bias (7–9 stars) [15].

### Outcomes and data synthesis

The primary outcomes were ED revisits and hospital readmissions after the index fall-related ED presentation. Outcomes were extracted as reported by study authors and standardised into ED revisits and hospital readmissions. ED revisits and hospital readmissions were considered all-cause unless studies explicitly reported fall-related events or revisits/readmissions related to a specific injury or diagnosis. Hospital readmission was defined as rehospitalisation after the index presentation, and terms such as ‘ED readmission’, ‘ED reattendance’ and ‘return visit’ were classified as ED revisits. Outcomes were included if reported within 1 year of follow-up and if sufficient data were available. Secondary objectives were to summarise determinants associated with ED revisits and hospital readmissions and to explore heterogeneity by patient population, care setting, outcome definition and follow-up duration.

Separate random-effects meta-analyses were stratified by follow-up interval (up to 1 year) and outcome. When multiple timepoints were reported, each contributed only to its corresponding interval-specific analysis. Proportions were pooled using logit transformation with inverse-variance weighting, and between-study variance (*τ*^2^) was estimated using restricted maximum likelihood. Results are presented as proportions with 95% confidence intervals (CIs) in forest plots, where pooled results were included in case of two or more available studies. Statistical heterogeneity was assessed using *I*^2^ and *τ*^2^. To aid interpretation of between-study heterogeneity and the potential variability of results across future comparable settings, 95% prediction intervals were calculated using the *metaprop* function from the *meta* package in *R* for meta-analyses including at least three studies. All analyses were performed in *R* (version 4.2.2).

### Sensitivity analyses and reporting bias

Robustness was assessed by leave-one-out analyses and by sensitivity analyses excluding studies at higher risk of bias (NOS ≤3 stars). Meta-regression and formal assessment of reporting bias were not done because of the small number of studies per outcome (<10), high heterogeneity and the limited interpretability of funnel plots for proportion meta-analyses.

### Results

The database search identified 4257 records. After removal of 1212 duplicates, 3045 titles and abstracts were screened, of which 223 articles underwent full-text assessment. Full texts could not be retrieved for 46 reports. Of the remaining 177 reports, 20 articles, representing 19 unique studies were included. Inter-rater reliability for full-text screening was high (Cohen’s κ = 0.84; proportion of agreement = 0.96). The study selection process is shown in Figure 1.

Studies were excluded mainly because they did not report ED revisits or hospital readmissions (*n* = 92). Studies reporting mortality were initially included at abstract screening, as these were considered likely to also report revisit and readmission outcomes, but were excluded at full-text review if such relevant outcomes were absent. Studies of non-fall-related index presentations and those without fall-specific subgroup analyses were also excluded.

### Study characteristics

A total of 20 articles representing 19 unique studies published between 2008 and 2025 were included [1–5, 16–30]. Two articles reported analyses from the same dataset and were combined [20, 21]. Study size ranged from 30 to 997 524 participants, with a total of 1 609 871 individuals. Most studies were retrospective cohort studies (*n* = 12) [1, 2, 5, 16, 17, 19, 21–25, 30], followed by prospective cohorts (*n* = 4) [3, 4, 26, 27], retrospective chart reviews (*n* = 2) [28, 29] and one nested case–control study [18]. The USA contributed 12 studies [1, 2, 4, 5, 17, 19, 21, 23, 25, 27–29], with additional studies from Europe (*n* = 4) [3, 18, 22, 24], Canada [16], Australia [26] and Singapore [30]. Nine studies were monocentric [3, 4, 17, 21, 24–27, 29], and 10 were multicentre or population-based [1, 2, 5, 16, 18, 19, 22, 23, 28, 30].

Most studies included adults aged 65 years or older (*n* = 15) [1, 2, 4, 5, 16, 17, 19, 21–25, 27–29], three studies used higher age thresholds [3, 18, 26] and one study included adults aged 55 years or older [30]. However, all reported mean or median ages of at least 70 years. Definitions of fall-related ED presentation varied and were based on administrative coding, clinical documentation or both. Some studies focused on specific fall subtypes, such as low-energy or same-level falls or injury-specific presentations. The full study characteristics summary is presented in Table 1.

**Table 1:** Characteristics of included studies reporting ED revisits and/or hospital readmissions within 1 year after an index fall-related ED presentation in older adults.

Study (year)	Country	Study design	Setting	Sample size	Population	Outcome and follow-up	Key results
Alvarez Avendano *et al.* (2025) [25]	USA	Retrospective cohort	Single-centre academic ED	1581	≥65 yrs, mean 80.6 (SD 8.9)	ED revisit and hospital readmission (all-cause), 30 days	ED revisit: 18.5%; hospital readmission: 3.2%
Close *et al.* (2012) [26]	Australia	Prospective cohort	Single-centre university hospital	1290	≥70 yrs, mean 82.5 (SD 6.9)	Hospital readmission (all-cause), 28 days	Hospital readmission: 13.4%
Cox *et al.* (2022) [2]	USA	Retrospective cohort	Multicentre state-wide EDs	206 612	≥65 yrs; 65–74 (35.5%), 75–84 (33.5%), ≥85 (31.0%)	ED revisit (all-cause), 7 days	ED revisit: 11.7%
Earl-Royal *et al.* (2017) [19]	USA	Retrospective cohort	State-wide administrative ED and inpatient databases	92 599	≥65 yrs, mean 77.3 (SD 8.3)	Hospital readmission (all-cause), 9 and 30 days	9-day: 4.8%; 30-day: 9.6%
El Abd *et al.* (2022) [18]	France	Retrospective nested case–control	Multicentre academic EDs	579	≥75 yrs, mean 86.1 (SD 6.0)	Hospital readmission (all-cause), 30 days	Hospital readmission: 17.8%
Farrell *et al*. (2023) [17]	USA	Retrospective cohort	Single-centre level I trauma centre	1087	≥65 yrs, mean 79.9	Hospital readmission (all-cause), 30 days	Hospital readmission: 12.4%
Fillion *et al.* (2019) [16]	Canada	Retrospective cohort	Population-based administrative database	178 304	≥65 yrs, mean 75.5 (SD 7.5)	ED revisit and hospital readmission (all-cause), 1 year	ED revisit: 44.8%; hospital readmission: 31.9%
Hosseinpour *et al.* (2022) [23]	USA	Retrospective cohort	Nationwide emergency and inpatient database	100 850	≥65 yrs, mean 82.2 (SD 9.7)	ED revisit (all-cause), 30 days	ED revisit: 11.5%
Kirchhoff *et al*. (2008) [24]	Denmark	Retrospective cohort	Single-centre ED	535	≥65 yrs; 65–79 (41%), ≥80 (59%)	ED revisit and hospital readmission (all-cause), 6 months	ED revisit: 28%; hospital readmission: 30%
Liu *et al.* (2015) [1]	USA	Retrospective cohort	Multicentre urban teaching hospitals	21 340	≥65 yrs, mean 79 (SD 8)	ED revisit (all-cause), 3 days, 7 days, 30 days and 1 year	3-day: 2%; 7-day: 5%; 30-day: 13%; 1-year: 25%
Sarwari *et al.* (2024) [22]	Denmark	Retrospective cohort	Regional administrative registries	605	≥65 yrs, mean 86.1 (SD 7.4)	ED revisit and hospital readmission (all-cause), 30 days	ED revisit: 13%; hospital readmission: 12%
Schrijver *et al.* (2013) [3]	Netherlands	Prospective cohort	Single-centre university medical centre	30	≥70 yrs, median 81 (range 70–97)	ED revisit (all-cause), 30 days	ED revisit: 13.3%
Selman *et al.* (2024) [27]	USA	Prospective cohort	Single-centre academic ED	309	≥65 yrs, mean 81 (range 65–100)	ED revisit (fall-related), 3, 6 and 12 months	3-month: 15.9%; 6-month: 22.7%; 12-month: 23.7%
Shankar *et al.* (2017) [4]	USA	Prospective cohort	Single-centre level I trauma centre	87	≥65 yrs, mean 72	ED revisit (fall-related), 60 days	ED revisit: 8.0%
Shankar *et al.* (2020) [5]	USA	Retrospective cohort	State-wide administrative ED and inpatient databases	997 524	≥65 yrs, mean 79.5 (SD 8.3)	ED revisit (all-cause), 7 days, 30 days, 6 months and 1 year	7-day: 12%; 30-day: 22%; 6-month: 43%; 1-year: 55%
Southerland *et al.* (2014) [29]	USA	Retrospective chart review	Single-centre academic ED	315	≥65 yrs, median 77 (IQR 69–83)	ED revisit (all-cause), 72 h	72-h: 6.3%
Southerland *et al.* (2016) [28]	USA	Retrospective chart review	Multicentre EDs	263	≥65 yrs, mean 76.8 (SD 8.6)	ED revisit (all-cause), 72 h, 30 days and 90 days	72-h: 5%; 30-day: 13%; 90-day: 22%
Sri-on *et al.* (2017) [21]	USA	Retrospective cohort	Single-centre academic ED	350	≥65 yrs, median 81 (IQR 15)	ED revisit and hospital readmission (all-cause), 30 days and 6 months	30-day ED revisit: 17.7%; 6-month ED revisit: 42.5%; 30-day readmission: 11.1%; 6-month readmission: 31.1%
Wong *et al.* (2019) [30]	Singapore	Retrospective cohort	National trauma registry (public hospitals)	5671	≥55 yrs, median 74 (IQR 64–83)	ED revisit (all-cause), 30 days and 1 year	30-day: 13.6%; 1-year: 42.3%

SD = standard deviation; IQR = interquartile range.

### Outcome measures and follow-up

Follow-up duration varied across studies. Six studies reported very short-term outcomes within 3–9 days [1, 2, 5, 19, 28, 29], 13 studies reported outcomes at 30 days [1, 3, 5, 17–19, 21–23, 25, 26, 28, 30], 3 studies reported intermediate follow-up at 60–90 days [4, 27, 28] and 7 studies reported longer-term outcomes at 6 or 12 months [1, 5, 16, 21, 24, 27, 30]. Ten studies reported ED revisits only [1–5, 23, 27–30], four reported hospital readmissions only [17–19, 26] and five reported both outcomes [16, 21, 22, 24, 25]. Most studies reported all-cause outcomes (*n* = 17), whereas two reported fall-specific outcomes [4, 27]. Because of differences in outcome definitions and follow-up intervals, fall-specific outcomes were not included in the pooled estimates.

### Risk of bias

Seven studies were classified as low risk of bias [2, 5, 16, 18, 22, 23, 30], nine as moderate risk of bias [1, 3, 17, 19, 21, 24, 25, 27, 28] and three as high risk of bias [4, 26, 29]. Limitations included restricted populations or monocentric designs, absence of non-exposed cohorts, limited adjustment for confounding and incomplete follow-up capture, which might have led to underestimation of outcomes. Full risk-of-bias assessments are provided in Supplementary Table S2.

### ED revisits

**Figure 2:**
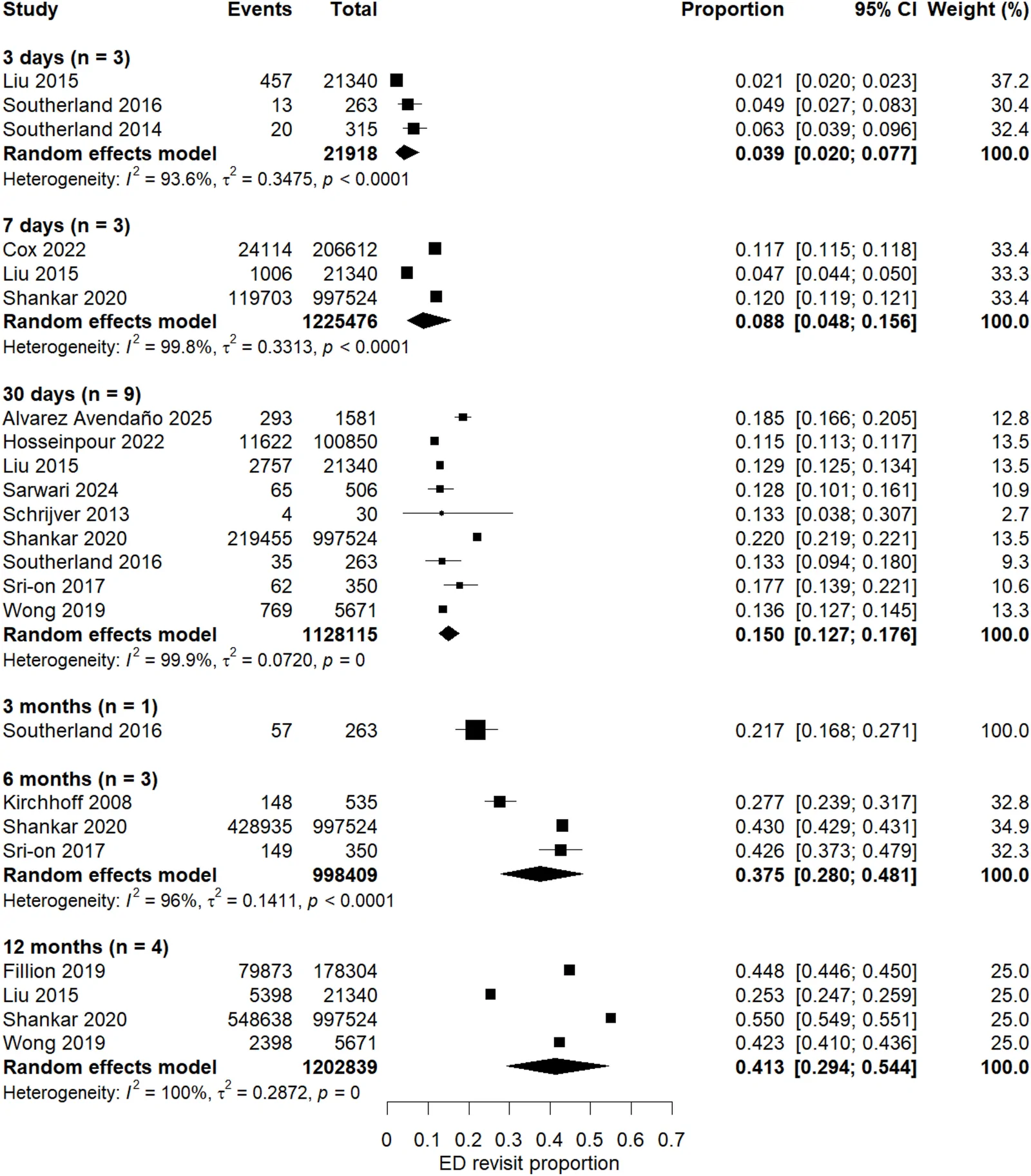
Random-effects meta-analysis of ED revisit proportions after an index fall-related ED presentation in older adults, stratified by follow-up interval. Estimates are pooled proportions with 95% CIs.

ED revisit outcomes were reported across six follow-up intervals within 12 months (Figure 2). Pooled proportions were higher with increasing follow-up duration, ranging from 3.9% within 3 days (*n* = 3; 95% CI 2.0–7.7) to 8.8% within 7 days (*n* = 3; 4.8–15.6), and 15.0% within 30 days (*n* = 9; 12.7–17.6). At longer follow-up intervals of 3, 6 and 12 months, proportions were 21.7% (*n* = 1; 16.8–27.1), 37.5% (*n* = 3; 28.0–48.1) and 41.3% (*n* = 4; 29.4–54.4), respectively.

### Hospital readmissions

**Figure 3:**
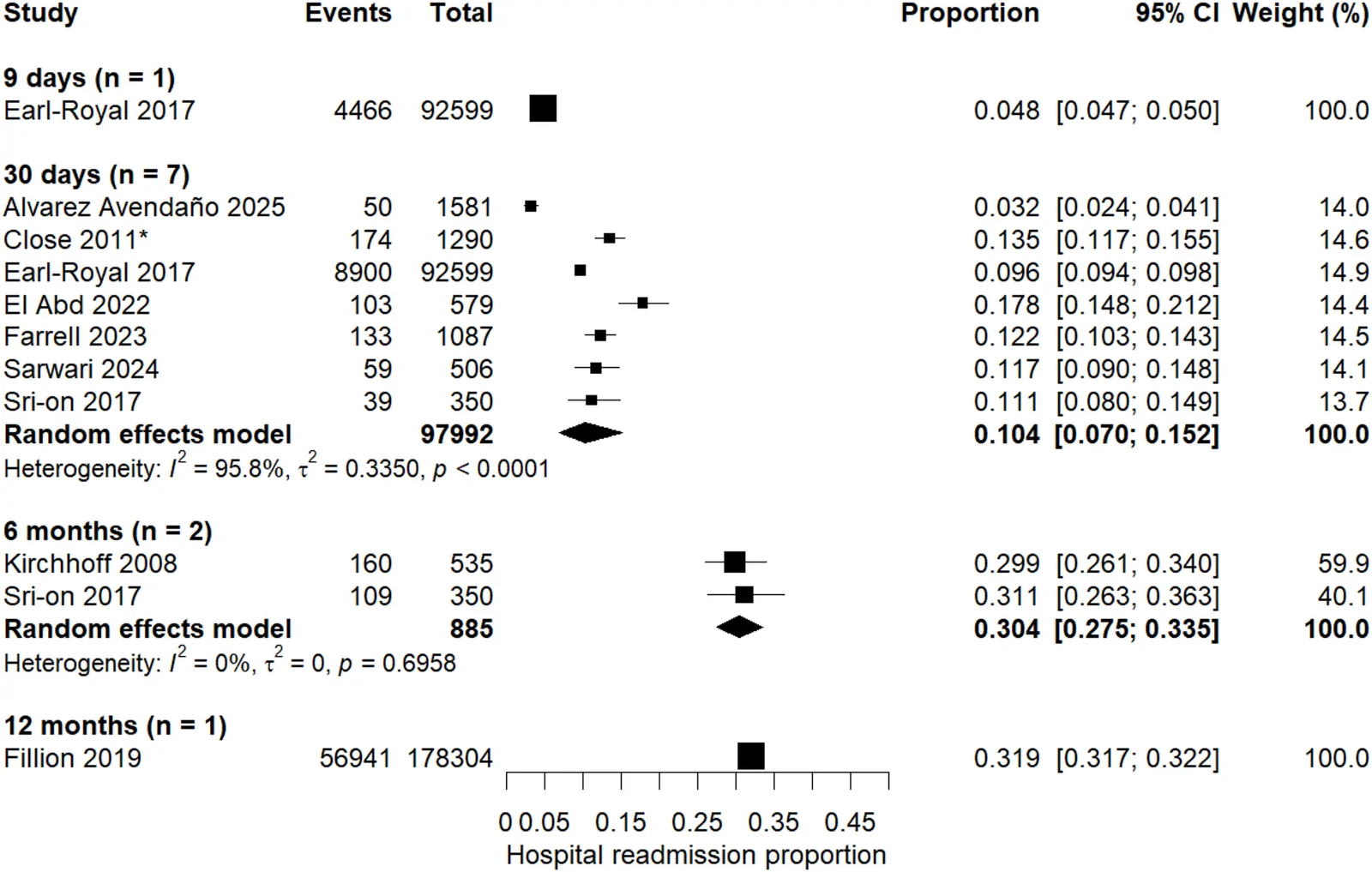
Random-effects meta-analysis of hospital readmission proportions after an index fall-related ED presentation in older adults, stratified by follow-up interval. Pooled estimates are shown for 30 days and 6 months; other intervals represent single-study estimates. Estimates are pooled proportions with 95% CIs unless otherwise indicated.

Hospital readmission data were available for fewer timepoints (Figure 3). At 9 days, a single study reported a proportion of 4.8% (95% CI 4.7–5.0). At 30 days (*n* = 7), the pooled proportion was 10.4% (7.0–15.2), with substantial heterogeneity (*I*^2^ = 95.8%; *τ*^2^ = 0.3350).

At 6 months (*n* = 2), the pooled proportion was 30.4% (27.5–33.5), with no observed heterogeneity (*I*^2^ = 0.0%; *τ*^2^ = 0) due to similar proportions within the two studies (29.9% and 31.1%). At 12 months, one study reported a proportion of 31.9% (31.7–32.2). Table 2 summarises pooled proportions, heterogeneity statistics and prediction intervals across follow-up intervals.

**Table 2:** Pooled proportions of ED revisits and hospital readmissions after an index fall-related ED presentation, by follow-up interval.

Time after index fall	Outcome	*n*		Pooled proportion (%)	95% CI	*I* ^2^ (%)	95% prediction interval
3 days	ED revisit	3		3.9	2.0–7.7	93.6	0.2–44.6
7 days	ED revisit	3		8.8	4.8–15.6	99.8	0.5–62.7
30 days	ED revisit	9		15.0	12.7–17.6	99.9	8.4–25.5
3 months	ED revisit	1		21.7	16.8–27.1	NA	NA
6 months	ED revisit	3		37.5	28.0–48.1	96.0	8.4–79.7
12 months	ED revisit	4		41.3	29.4–54.4	100.0	9.5–82.6
9 days	Hospital readmission	1		4.8	4.7–5.0	NA	NA
30 days	Hospital readmission	7		10.4	7.0–15.2	95.8	2.5–34.6
6 months	Hospital readmission	2		30.4	27.5–33.5	0.0	NA
12 months	Hospital readmission	1		31.9	31.7–32.2	NA	NA

*n* = number of studies contributing to the estimate. Estimates based on a single study were not pooled. Prediction intervals were calculated only for pooled estimates based on three or more studies and indicate the range within which the true proportion of a future comparable study is expected to fall, accounting for between-study heterogeneity.

### Heterogeneity and uncertainty

Substantial heterogeneity was observed across most pooled analyses. For ED revisits, *I*^2^ values ranged from 93.6% to 100.0%, indicating substantial between-study variability. Similarly, heterogeneity was substantial for 30-day hospital readmissions (*I*^2^ = 95.8%; *τ*^2^ = 0.3350). Corresponding 95% prediction intervals were generally substantially wider than the confidence intervals, reflecting the relatively large between-study heterogeneity observed across analyses (Table 2). For example, the pooled 30-day ED revisit proportion was 15.0% (95% CI 12.7–17.6), whereas the corresponding 95% prediction interval ranged from 8.4% to 25.5%. Likewise, the pooled 12-month ED revisit proportion was 41.3% (95% CI 29.4–54.4), with a prediction interval ranging from 9.5% to 82.6%. For 30-day hospital readmissions, the pooled proportion was 10.4% (95% CI 7.0–15.2), with a prediction interval ranging from 2.5% to 34.6%.

### Sensitivity analyses

Sensitivity analyses showed that pooled estimates were robust. Excluding studies at high risk of bias resulted in minimal changes (e.g. ED revisits at 3 days 3.9% to 3.1%; 30-day readmissions 10.4% to 9.9%). Leave-one-out analyses showed similar stability, with 30-day ED revisit estimates ranging from 14.1% to 15.6% and readmission estimates from 9.4% to 12.4%. Greater variability was observed at early timepoints because of the small number of studies. Full results are provided in Supplementary Tables S3–S5).

### Associated factors

Eleven studies reported independent associations between patient characteristics and subsequent ED revisits or hospital readmissions [1, 2, 16–19, 21–23, 28, 30], whereas the remaining studies either did not perform formal predictor analyses or identified no independent associations (Supplementary Table S6). Frailty, comorbidity burden, polypharmacy, male sex and neuropsychiatric factors were among the most frequently reported factors associated with recurrent healthcare utilisation. Several other factors were evaluated less frequently or yielded inconsistent findings across studies. Overall, substantial heterogeneity in the factors assessed, outcome definitions, follow-up periods and analytical approaches limited comparability and precluded formal quantitative synthesis.

### All-cause versus fall-specific outcomes

Four studies reported both all-cause and fall-specific outcomes [1, 22, 23, 30], while two studies reported fall-specific outcomes only [4, 27]. Across these studies, fall-specific outcomes were generally lower than all-cause ED revisits and hospital readmissions. In Liu *et al*., all-cause ED revisit rates increased from 2.0% at 3 days to 25.0% at 1 year, whereas corresponding fall-specific revisits increased from only 0.8% to 7.3% [1]. Hosseinpour *et al*. reported a 30-day fall-related ED revisit rate of 2.0%, and Wong *et al*. found that only 2.5% of patients experienced a repeat injurious fall during 12 months of follow-up [23, 30]. Sarwari *et al*. also observed lower trauma-related than all-cause acute hospital referrals, although the difference was less pronounced [22]. Similarly, fall-specific revisit rates reported by Selman *et al*. and Shankar *et al*. remained below our pooled all-cause estimates despite longer follow-up periods [4, 27]. Given the substantial differences in outcome definitions and follow-up periods, fall-specific outcomes were interpreted separately and were not pooled.

### Discussion

In this systematic review and meta-analysis, we quantified recurrent acute care use after an index fall-related ED presentation in adults aged 65 years and older. Both ED revisits and hospital readmissions were common with cumulative proportions increasing across longer follow-up intervals. Together, these findings support the interpretation that recurrent acute care use following a fall-related ED presentation often reflects broader vulnerability and ongoing healthcare needs rather than representing an isolated acute episode.

This temporal pattern is clinically important. The accumulation of revisits and readmissions across the first year suggests that recurrent acute care use is not limited to the immediate consequences of the index fall but may instead reflect broader vulnerability and underlying health decline. In the context of healthy ageing, recurrent acute care use after a fall can be understood as a downstream manifestation of reduced resilience, frailty and progressive loss of intrinsic capacity [31, 32]. Consistent with the World Falls Guidelines, falls may therefore be considered not only events but also as clinical manifestations of underlying pathology [2, 8, 33–35]. In this context, a fall-related ED presentation may serve as an observable clinical signal of underlying health decline.

Our pooled 30-day ED revisit estimate of 15.0% is consistent with individual cohort studies, but more importantly, this review is the first to quantify the burden across multiple timepoints up to 1 year. This longer horizon matters for both clinical practice and health systems, because it challenges the narrow focus on immediate injury management and highlights the substantial cumulative burden of recurrent acute care use, following a fall-related ED presentation [8, 12, 33, 35]. It also suggests that conventional short-term quality indicators might underestimate the true burden that follows fall-related ED presentation in older adults.

The high *I*^2^ values observed across analyses, together with substantial between-study variance (*τ*^2^), indicate that heterogeneity reflects meaningful differences in study populations, care contexts and follow-up capture rather than random variation alone. Potential sources of heterogeneity include differences in study populations, healthcare settings, definitions of fall-related ED presentation, outcome definitions and follow-up duration. This interpretation is further supported by the wide 95% prediction intervals observed across most analyses. While confidence intervals quantify the precision of the pooled estimates, prediction intervals reflect the range of event rates expected in future comparable settings. The substantial width of these intervals indicates that revisit and readmission rates may vary considerably across healthcare systems and patient populations, even when pooled estimates appear relatively precise. Rather than weakening the findings, this heterogeneity underscores that post-fall trajectories are not uniform but vary according to patient characteristics, healthcare organisation and study context. Consequently, pooled estimates should be interpreted as averages across heterogeneous populations and healthcare settings, rather than as the expected event rate in any individual healthcare setting.

Several factors were repeatedly associated with ED revisits and hospital readmissions despite differences in study settings. Recurrent acute care use clustered around markers of frailty and comorbidity burden, with graded associations observed across indices [1, 2, 16, 19, 23, 28, 30]. Polypharmacy and the use of high-risk medications were identified as factors associated with ED revisits and readmissions [17, 21]. Male sex was associated with higher risk in several studies [1, 2, 18, 22, 30]. Although individual studies varied in design, outcome definitions, follow-up periods and the specific factors assessed, a broadly consistent pattern emerged across the available evidence. At the same time, many factors were evaluated in only a limited number of studies, precluding formal quantitative synthesis. Together, these findings suggest that recurrent healthcare utilisation following a fall frequently occurs in the context of broader vulnerability, frailty, multimorbidity and medication-related risk [36, 37].

Importantly, available evidence suggests that uptake of recommended follow-up after ED presentation, such as comprehensive geriatric assessment (CGA) or multifactorial fall risk assessment, remains suboptimal, although data are limited [9, 11, 38–40]. As a result, potentially modifiable contributors, including unrecognised comorbidities, adverse drug effects and functional impairments, may remain insufficiently identified and addressed. Although our review was not designed to evaluate the impact of follow-up care on recurrent healthcare utilisation, these observations highlight potential areas for future investigation.

The findings of this study have implications for clinical practice. Older adults presenting after a fall are at substantial risk of subsequent ED use and hospitalisation, even when the initial injury appears minor. The index ED presentation may therefore provide an opportunity to identify frailty and modifiable underlying risk factors, including undiagnosed disease, medication-related harm and functional decline. In line with current guideline recommendations, including the World Falls Guidelines, multifactorial fall risk assessment and broader geriatric evaluation may be considered where feasible [33]. However, comprehensive geriatric interventions can increase ED length of stay and have shown inconsistent effects on revisits and readmissions [38, 41, 42]. Targeted interventions that can be integrated into routine ED workflows, such as medication review and pragmatic frailty-based risk stratification, may be more compatible with the operational realities of the ED, although evidence for their effectiveness remains limited [43, 44].

Bridging the gap between guideline recommendations and clinical practice remains a key challenge [35]. Existing ED-initiated fall prevention pathways and integrated geriatric care models provide a conceptual framework, but robust evidence that they reduce recurrent acute care use is still limited [11, 38, 42]. At the health-system level, pathways linking acute care with geriatric assessment, medication optimisation, fall prevention and community-based support may better address the needs of older adults than ED-only management, although direct evidence remains limited [33, 35, 45]. These findings highlight the need for implementation-focused research to translate evidence-based recommendations into feasible, sustainable and scalable models of care. Future research should also aim to better understand the mechanisms underlying recurrent healthcare utilisation following a fall-related ED presentation, distinguish fall-related from broader frailty-related drivers of healthcare use and evaluate feasible models of care for this population.

This review has several strengths. To our knowledge, it is the first systematic review and meta-analysis to estimate pooled proportions of both ED revisits and hospital readmissions after an index fall-related ED presentation in older adults across multiple follow-up intervals up to 1 year. Additional strengths include a comprehensive search strategy without language restrictions, inclusion of studies from diverse settings and the use of random-effects models together with 95% prediction intervals to aid interpretation of the substantial between-study heterogeneity. The review also has limitations, most of which reflect the available evidence base. Included studies were highly heterogeneous in design, populations, outcome definitions and follow-up capture, resulting in substantial clinical and statistical heterogeneity. Many studies relied on retrospective data sources with limited adjustment for confounding, and key constructs such as frailty, medication use and multimorbidity were not measured consistently. Estimates at some longer-term timepoints were based on relatively few studies, limiting precision. Furthermore, only a subset of included studies evaluated factors associated with recurrent healthcare utilisation, limiting the strength of conclusions regarding potential predictors. Most included studies lacked non-fall comparison groups. Consequently, the available evidence remains insufficient to determine the extent to which recurrent healthcare utilisation is attributable to falls themselves rather than to the broader vulnerability of older adults presenting after a fall. Because this review synthesised observational evidence, causal inference is limited and residual bias remains possible despite conservative risk-of-bias assessment and sensitivity analyses. Generalisability might also be limited by the predominance of studies from high-income countries.

In summary, recurrent ED visits and hospital readmissions after a fall are common in older adults, with cumulative proportions increasing across longer follow-up intervals. Approximately 9% of patients revisit the ED within 7 days, rising to 15% within 30 days and over 40% within 12 months. Hospital readmissions follow a similar longer-term pattern. Frailty, comorbidity burden and medication-related factors were among the most consistently reported factors associated with recurrent healthcare utilisation. Recognising a fall as a potential clinical expression of broader vulnerability and underlying health decline, rather than an isolated event, may help inform future approaches to risk assessment, follow-up and care delivery for older adults presenting to the ED.

## Supplementary Material

Supplementary_materials_afag271

## Data Availability

This review is based on previously published studies, so all data used are publicly available. Relevant study data are included in the article or provided as supplementary information.
