# Supplementary material for: Emergency department revisits and hospital readmissions following a fall-related emergency department presentation in older adults: a systematic review and meta-analysis
Source: Age Ageing. 2026 Sep 14;55(9):afag271. doi: 10.1093/ageing/afag271 (PMC13574242; doi:10.1093/ageing/afag271)
Supplement: Supplementary file 1 [file supplementary_materials_afag271.pdf]

Emergency Department Revisits and Hospital Readmissions Following a Fall-Related Emergency Department Presentation in Older Adults: A Systematic Review and Meta-Analysis

Table of Contents

Supplementary Table S1. Search strategy 2

Supplementary Table S2. Risk-of-bias assessment of included studies using the Newcastle-Ottawa Scale 3

Supplementary Table S3. Sensitivity analyses excluding studies at high risk of bias 5

Supplementary Table S4. Leave-one-out sensitivity analyses for emergency department revisits 6

Supplementary Table S5. Leave-one-out sensitivity analyses for hospital readmissions 7

Supplementary Table S6. Overview of factors associated with ED revisits and hospital readmissions after a fall-related ED presentation 8

Supplementary Table S7. PRISMA 2020 checklist 10

Supplementary Table S1. Search strategy

Database	Search strategy	Records retrieved
PubMed/ MEDLINE	(("Accidental Falls"[Mesh] OR fall*[tiab])) AND ("Emergency Service, Hospital"[Mesh] OR ED[tiab] OR "Emergency Medical Services"[Mesh] OR emergency department[tiab] OR emergency room[tiab] OR acute care[tiab] OR "Accident and Emergency"[tiab] OR "Emergency care unit"[tiab] OR "emergency care"[tiab] OR ER[tiab]) AND ("Aged"[Mesh] OR "older adults"[tiab] OR "older people"[tiab] OR "older persons"[tiab] OR elderly[tiab] OR "geriatric patients"[tiab]) AND (ED revisit*[tiab] OR "emergency department revisit*"[tiab] OR "return visit*"[tiab] OR reattendance[tiab] OR readmission[tiab] OR hospitalization[tiab] OR rehospitalization[tiab] OR "Mortality"[Mesh] OR death*[tiab] OR recidivism[tiab] OR return*[tiab])	1176<br>10-11-2025
Embase	1 fall*.ti,ab. 370992<br>2 falling/ or exp fall-related injury/ 57067<br>3 fall-related injury/ 140<br>4 1 or 2 or 3 392313<br>5 emergency ward/ or emergency health service/ 375444<br>6 (acute adj care).ti,ab. 45798<br>7 ED.ti,ab. 172410<br>8 5 or 6 or 7 507067<br>9 aged/ 4380444<br>10 (older adj1 adult*).ti,ab. 203542<br>11 (older adj1 person*).ti,ab. 20245<br>12 (older adj1 people).ti,ab. 56012<br>13 (geriatric adj1 patient*).ti,ab. 16893<br>14 9 or 10 or 11 or 12 or 13 4453800<br>15 ED revisit*.ti,ab. 886<br>16 (emergency adj department adj revisit*).ti,ab. 230<br>17 (return adj visit*).ti,ab. 3099<br>18 recidivism.ti,ab. 6552<br>19 return*.ti,ab. 450725<br>20 reattendance.ti,ab. 323<br>21 hospital readmission/ 127603<br>22 mortality/ or death/ or all cause mortality/ or mortality rate/ 1488225<br>23 15 or 16 or 17 or 18 or 19 or 20 or 21 or 22 2013125<br>24 4 and 8 14827<br>25 14 and 24 5753<br>26 23 and 25 1536	1536<br>09-11-2025
Scopus	(TITLE-ABS-KEY ("accidental falls" OR fall*)) AND (TITLE-ABS-KEY ("emergency department" OR "emergency room" OR "emergency medical services" OR "emergency care unit" OR "emergency care" OR "accident and emergency" OR "acute care" OR ED OR ER)) AND (TITLE-ABS-KEY ("older adults" OR "older people" OR "older persons" OR elderly OR "geriatric patients")) AND (TITLE-ABS-KEY ("ED revisit*" OR "emergency department revisit*" OR "return visit*" OR reattendance OR readmission OR hospitalization OR rehospitalization OR death* OR mortality OR recidivism OR return*)) 1	1561<br>10-11-2025

Search date is shown below each database strategy. The term “Aged” was removed from the Scopus search because it retrieved a high number of irrelevant records.

Supplementary Table S2. Risk-of-bias assessment of included studies using the Newcastle-Ottawa Scale

	Selection				Comparability		Outcome			Total score	
**Publication**	**Represen-tativeness of the exposed cohort**	**Selection of the non-exposed cohort**	**Ascertainment of exposure**	**Demonstration outcome not present at start**	**Comparability**	**Comparability (additional factors)**	**Assess-ment of outcome**	**Was follow-up long enough for outcomes to occur**	**Adequacy of follow-up of cohorts**	**Total score**	**Overall risk of bias**
El Abd et al., 2022	★	★	★	★	★	★	★	★		8	Low
Farrell et al., 2023	★		★	★	★		★	★		6	Moderate
Sri-on et al. , 2017			★	★	★		★	★		5	Moderate
Alvarez Avendaño et al., 2025		★	★	★	★		★	★		6	Moderate
Selman et al., 2024			★	★			★	★		4	Moderate
Southerland et al., 2016			★	★	★		★	★		5	Moderate
Close et al., 2011			★				★	★		3	High
Wong et al., 2019			★	★	★	★	★	★	★	7	Low
Southerland et al., 2014			★	★			★			3	High
Hosseinpour et al., 2022	★	★	★	★	★	★	★	★	★	9	Low
Shankar et al., 2020	★	★	★	★			★	★	★	7	Low
Earl-Royal et al., 2017			★	★	★		★	★	★	6	Moderate
Shankar et al., 2017			★	★			★			3	High
Cox et al., 2022	★	★	★	★	★		★		★	7	Low
Liu et al., 2015	★		★	★	★		★	★		6	Moderate

Fillion et al., 2019	★	★	★	★	★	★	★	★	8	Low
Schrijver et al., 2013			★	★	★	★	★		5	Moderate
Sarwari et al., 2024		★	★	★	★	★	★	★	7	Low
Kirchhoff et al., 2008			★	★		★	★	★	5	Moderate

NOS = Newcastle-Ottawa Scale. Studies were classified as high risk of bias (0-3 stars), moderate risk of bias (4-6 stars), and low risk of bias (7-9 stars).

Supplementary Table S3. Sensitivity analyses excluding studies at high risk of bias

Outcome	Timepoint	Primary estimate (95% CI)	Number of studies (primary)	Number of studies (after exclusion)	Estimate after exclusion (95% CI)
ED revisit	3 days	0.039 (0.02–0.077)	3	2	0.031 (0.014–0.070)
Hospital readmission	30 days	0.104 (0.07–0.152)	7	6	0.099 (0.062–0.154)

Sensitivity analyses excluding studies at high risk of bias for pooled proportions of emergency department revisits and hospital readmissions after an index fall-related emergency department presentation. Estimates represent pooled proportions from the primary meta-analysis and after exclusion of studies classified as high risk of bias.

Supplementary Table S4. Leave-one-out sensitivity analyses for emergency department revisits

Timepoint	Primary estimate (95% CI)	LOO range	Most influential study
3 days	0.039 (0.02–0.077)	0.031–0.058	Liu 2015
7 days	0.088 (0.048–0.156)	0.075–0.118	Liu 2015
30 days	0.15 (0.127–0.176)	0.141–0.156	Shankar 2020
6 months	0.375 (0.280–0.481)	0.347–0.430	Kirchhoff 2008
12 months	0.413 (0.294–0.544)	0.370–0.474	Liu 2015

Leave-one-out sensitivity analyses for pooled proportions of emergency department revisits after an index fall-related emergency department presentation. Estimates represent pooled proportions from the primary meta-analysis, with ranges indicating variation after sequential exclusion of individual studies. The most influential study is defined as the study whose exclusion resulted in the largest change in the pooled estimate.

Supplementary Table S5. Leave-one-out sensitivity analyses for hospital readmissions

Timepoint	Primary estimate (95% CI)	LOO range	Most influential study
30 days	0.104 (0.07–0.152)	0.094–0.124	Alvarez Avendaño 2025
6 months	0.304 (0.275–0.335)	0.299–0.311	Kirchhoff 2008

Leave-one-out sensitivity analyses for pooled proportions of hospital readmissions after an index fall-related emergency department presentation. Estimates represent pooled proportions from the primary meta-analysis, with ranges indicating variation after sequential exclusion of individual studies. The most influential study is defined as the study whose exclusion resulted in the largest change in the pooled estimate.

Supplementary Table S6. Overview of factors associated with ED revisits and hospital readmissions after a fall-related ED presentation

Study	Outcome	Factors assessed	Factors independently associated with the outcome
Sri-on et al. 2017	ED revisit / Hospital readmission	Demographics, falls history, fracture history, medication use	Psychoactive or sedative medication use
Fillion et al. 2019	ED revisit / Hospital readmission	Frailty (ERA score)	Increasing frailty level (ERA score)
Cox et al. 2022	ED revisit	Demographics, comorbidity burden, psychiatric disease, substance use	Older age, male sex, ethnicity, higher Charlson Comorbidity Index, psychiatric disease, alcohol/substance use disorder
Hosseinpour et al. 2022	Hospital readmission	Frailty (Modified Frailty Index)	Modified Frailty Index >0.4
El Abd et al. 2022	Hospital readmission	Demographics, laboratory parameters, injury characteristics	Male sex, traumatic injury, elevated CRP, anaemia
Liu et al. 2015	ED revisit	Demographics, comorbidity burden, healthcare access, index-visit characteristics	Older age, male sex, Hispanic ethnicity, higher Charlson Comorbidity Index, no primary care physician, admission during index visit, socioeconomic factors
Wong et al. 2019	Hospital readmission	Injury severity, frailty (Modified Frailty Index), comorbidity burden, demographics, discharge destination	Older age, male sex, frailty (MFI), comorbidity burden, injury severity, discharge destination
Farrell et al. 2023	Hospital readmission	Demographics, medication use, comorbidity burden	Older age, hyperlipidaemia, polypharmacy (≥ 4 medications), antihypertensive use
Sarwari et al. 2024	Hospital readmission	Demographics, index disposition	Male sex, treat-and-release ED visit
Southerland et al. 2016	ED revisit	Comorbidity burden, injury characteristics	Higher Charlson Comorbidity Index, head trauma
Earl-Royal et al. 2017	Hospital readmission	Demographics, socioeconomic status, injury characteristics, chronic conditions	Number of chronic conditions, injury severity, socioeconomic factors

Schrijver et al., 2013; Alvarez Avendaño et al., 2025; Close et al., 2011; Shankar et al., 2017; Shankar et al., 2020; Sri-on et al., 2016; Kirchhoff et al., 2008; Selman et al., 2024; Southerland et al., 2014	ED revisit and/or hospital readmission	Various	No formal predictor analysis performed, insufficient data available for analysis, or no independent associations reported

Footnote: Factors assessed, outcome definitions, follow-up periods, and analytical approaches varied substantially across studies. Many factors were evaluated in only a single study and several findings were inconsistent across studies. Consequently, formal quantitative synthesis of associated factors was not considered appropriate and findings should be interpreted descriptively.

Supplementary Table S7. Preferred Reporting Items for Systematic reviews and Meta-Analyses-network meta-analysis (PRISMA) checklist.

Section and Topic	Item #	Checklist item	Location where item is reported
**TITLE**			
Title	1	Identify the report as a systematic review.	Title
**ABSTRACT**			
Abstract	2	See the PRISMA 2020 for Abstracts checklist.	Check (=summary)
**INTRODUCTION**			
Rationale	3	Describe the rationale for the review in the context of existing knowledge.	Paragraphs 1-3
Objectives	4	Provide an explicit statement of the objective(s) or question(s) the review addresses.	End of introduction
**METHODS**			
Eligibility criteria	5	Specify the inclusion and exclusion criteria for the review and how studies were grouped for the syntheses.	Methods- prospective and retrospective....
Information sources	6	Specify all databases, registers, websites, organisations, reference lists and other sources searched or consulted to identify studies. Specify the date when each source was last searched or consulted.	Search paragraph (PubMed, Embase, Scopus, Nov 2025)
Search strategy	7	Present the full search strategies for all databases, registers and websites, including any filters and limits used.	Appendix Table S1
Selection process	8	Specify the methods used to decide whether a study met the inclusion criteria of the review, including how many reviewers screened each record and each report retrieved, whether they worked independently, and if applicable, details of automation tools used in the process.	Methods (records were imported into covidence and flowchart)
Data collection process	9	Specify the methods used to collect data from reports, including how many reviewers collected data from each report, whether they worked independently, any processes for obtaining or confirming data from study investigators, and if applicable, details of automation tools used in the process.	Methods (data extraction and risk of bias paragraph
Data items	10a	List and define all outcomes for which data were sought. Specify whether all results that were compatible with each outcome domain in each study were sought (e.g. for all measures, time points, analyses), and if not, the methods used to decide which results to collect.	Methods – outcomes and data synthesis paragraph
	10b	List and define all other variables for which data were sought (e.g. participant and intervention characteristics, funding sources). Describe any assumptions made about any missing or unclear information.	Data extraction paragraph
Study risk of bias assessment	11	Specify the methods used to assess risk of bias in the included studies, including details of the tool(s) used, how many reviewers assessed each study and whether they worked independently, and if applicable, details of automation tools used in the process.	Methods – NOS paragraph (and Table S2)
Effect measures	12	Specify for each outcome the effect measure(s) (e.g. risk ratio, mean difference) used in the synthesis or presentation of results.	Methods (proportions were pooled) paragraph
Synthesis methods	13a	Describe the processes used to decide which studies were eligible for each synthesis (e.g. tabulating the study intervention characteristics and comparing against the planned groups for each synthesis (item #5)).	Methods – grouping by outcome/follow-up
	13b	Describe any methods required to prepare the data for presentation or synthesis, such as handling of missing summary statistics, or data conversions.	Derivation of counts, (methods)
	13c	Describe any methods used to tabulate or visually display results of individual studies and syntheses.	See methods/figures/tables
	13d	Describe any methods used to synthesize results and provide a rationale for the choice(s). If meta-analysis was performed, describe the model(s), method(s) to identify the presence and extent of statistical heterogeneity, and software package(s) used.	Methods – random effects, I^2, τ^2, R
	13e	Describe any methods used to explore possible causes of heterogeneity among study results (e.g. subgroup analysis, meta-regression).	Discussed in methods

Section and Topic	Item #	Checklist item	Location where item is reported
	13f	Describe any sensitivity analyses conducted to assess robustness of the synthesized results.	Sensitivity pargraph
Reporting bias assessment	14	Describe any methods used to assess risk of bias due to missing results in a synthesis (arising from reporting biases).	Explicitely stated not performed (methods)
Certainty assessment	15	Describe any methods used to assess certainty (or confidence) in the body of evidence for an outcome.	Certainty of evidence (methods)
**RESULTS**			
Study selection	16a	Describe the results of the search and selection process, from the number of records identified in the search to the number of studies included in the review, ideally using a flow diagram.	Results – first paragraph, Figure 1
	16b	Cite studies that might appear to meet the inclusion criteria, but which were excluded, and explain why they were excluded.	Second paragraph
Study characteristics	17	Cite each included study and present its characteristics.	Results – Study characteristics and Table 1
Risk of bias in studies	18	Present assessments of risk of bias for each included study.	Results – Risk of Bias + Appendix
Results of individual studies	19	For all outcomes, present, for each study: (a) summary statistics for each group (where appropriate) and (b) an effect estimate and its precision (e.g. confidence/credible interval), ideally using structured tables or plots.	Table 1 + text
Results of syntheses	20a	For each synthesis, briefly summarise the characteristics and risk of bias among contributing studies.	Results – narrative/RoB section
	20b	Present results of all statistical syntheses conducted. If meta-analysis was done, present for each the summary estimate and its precision (e.g. confidence/credible interval) and measures of statistical heterogeneity. If comparing groups, describe the direction of the effect.	ED-revisits/readmissions paragraph + figures
	20c	Present results of all investigations of possible causes of heterogeneity among study results.	Not performed (random effects)
	20d	Present results of all sensitivity analyses conducted to assess the robustness of the synthesized results.	Results – sensitivity analysis paragraph and Table S3-S5
Reporting biases	21	Present assessments of risk of bias due to missing results (arising from reporting biases) for each synthesis assessed.	Not assessed (but mentioned in methods)
Certainty of evidence	22	Present assessments of certainty (or confidence) in the body of evidence for each outcome assessed.	Limited qualitative only, not per outcome (but see sensitibvity analysis)
**DISCUSSION**			
Discussion	23a	Provide a general interpretation of the results in the context of other evidence.	Par 1-4 discussion
	23b	Discuss any limitations of the evidence included in the review.	Limitations paragraph
	23c	Discuss any limitations of the review processes used.	General
	23d	Discuss implications of the results for practice, policy, and future research.	Clinical and system implications
**OTHER INFORMATION**			
Registration and protocol	24a	Provide registration information for the review, including register name and registration number, or state that the review was not registered.	Methods – PROSPERO
	24b	Indicate where the review protocol can be accessed, or state that a protocol was not prepared.	Link / registration number PROSPERO
	24c	Describe and explain any amendments to information provided at registration or in the protocol.	N/A
Support	25	Describe sources of financial or non-financial support for the review, and the role of the funders or sponsors in the review.	Funding statement (=none)
Competing interests	26	Declare any competing interests of review authors.	Declaration interests (none)

Section and Topic	Item #	Checklist item	Location where item is reported
Availability of data, code and other materials	27	Report which of the following are publicly available and where they can be found: template data collection forms; data extracted from included studies; data used for all analyses; analytic code; any other materials used in the review.	Data sharing = >
